# Pleiotropic Effects of Heparins: From Clinical Applications to Molecular Mechanisms in Hepatocellular Carcinoma

**DOI:** 10.1155/2018/7568742

**Published:** 2018-10-22

**Authors:** Peyda Korhan, Yeliz Yılmaz, Ezgi Bağırsakçı, Ayşim Güneş, Hande Topel, Brian I. Carr, Neşe Atabey

**Affiliations:** ^1^Izmir Biomedicine and Genome Center, 35340, Turkey; ^2^Medical Biology and Genetics, Heath Sciences Institute, Dokuz Eylul University, 35340, Turkey; ^3^Izmir International Biomedicine and Genome Institute, Dokuz Eylul University, 35340, Turkey

## Abstract

Hepatocellular carcinoma (HCC) is a major health problem worldwide and most cases are incurable because of late presentation. It is the most common primary neoplasm of the liver and often arises in the context of a chronic liver disease that impairs coagulation. Portal vein thrombosis (PVT) is a common complication of HCC that is associated with a poor prognosis. Heparin derivatives are widely used in the management of venous thromboembolism (VTE). Among them low molecular weight heparin (LMWH) favorably influences the survival in patients with advanced cancer, including HCC. Due to their pleiotropic function, heparins affect tumorigenesis in many ways and may promote or hamper tumorigenic transformation depending on the cancer type and cancer stage along with their structural properties and concentration. Thus, their application as an antithrombotic along with the conventional therapy regime should be carefully planned to develop the best management strategies. In this review, we first will briefly review clinical applications of heparin derivatives in the management of cancer with a particular focus on HCC. We then summarize the state of knowledge whereby heparin can crosstalk with molecules playing a role in hepatocarcinogenesis. Lastly, we highlight new experimental and clinical research conducted with the aim of moving towards personalized therapy in cancer patients at risk of thromboembolism.

## 1. Introduction

Worldwide, liver cancer is the sixth most common cancer and is the second leading global cause of cancer-related deaths [1, 2]. Among all primary liver cancers, HCC is the most prevalent malignancy, accounting for approximately 90% of cases [1–3]. The prognosis for HCC is very poor, with an incidence rate almost equaling the mortality rate (overall ratio of mortality to incidence of 0.95) [1]. The incidence of HCC increases progressively worldwide with advancing age in all populations, reaching a peak at 70 years [1]. The highest incidence and mortality rates of HCC are found in the less developed regions of the world, such as Southeast Asian countries [1], as compared to those in more developed regions in Europe and North America [1–3]. This global variation in incidence rates of HCC is closely related to the risk factors for HCC. HCC is common in patients with advanced hepatic fibrosis and cirrhosis, particularly with chronic damage caused by HBV or HCV infection, alcohol abuse, metabolic disease, and nonalcoholic fatty liver disease/obesity [4]. Tobacco smoke inhalation and dietary ingestion of fungal aflatoxins have been also recognized as major risk factors for HCC [4]. HCC originates as a result of an accumulation of genetic and epigenetic alterations, leading to an aberrant production of driver molecules. These altered expression profiles result in a multistep progression of precursor lesions to invasive/advanced HCC [4]. Importantly, Cancer Research, UK, has announced that 49% of liver cancer cases in the UK are preventable [5]. HBV vaccination, the establishment of treatments for HBV or HCV infections and widespread screening for hepatitis B or C viral infection, and interrupting the transmission of hepatitis virus infection via blood transfusion and blood products have all been shown to prevent liver cancer in high-incidence countries such as Japan [1, 5, 6].

Advances in science and technology have resulted in substantial opportunities for the management of HCC; however, prognosis of this disease is still poor due to the advanced stage at disease presentation, often due to absence of pathognomonic symptoms [7–10]. Larger tumor size, vascular invasion, poor liver functional status, and nodal metastasis are all associated with a poor prognosis [7–10]. Additionally, extraordinary inter- and intratumor heterogeneity of HCC contribute to drug resistance and recurrence, which pose a substantial bar to survival [11].

This complexity of HCC has led to the development of staging systems which combine both tumor and liver factors and a set of management guidelines, such as the Barcelona Clinic Liver Cancer (BCLC) guidelines recommended by the American Association for the Study of Liver Diseases (AASLD) and the European Association for the Study of the Liver (EASL) [8–10]. Surgical resection, transplantation, and ablation are potentially curative treatment options for HCC [8–10]. Unfortunately, less than 30% of patients globally who are diagnosed with early stage disease (Stage 0 or A) are eligible for these procedures [8–10]. For patients with intermediate stage disease (stage B), where patients are not eligible to be operated on, transarterial chemoembolisation (TACE) or transarterial radioembolisation (TARE) are recommended to establish local control and palliation [8–10]. TACE could potentially also be an adjuvant therapy for resectable HCC patients after hepatectomy, which could prevent recurrence and improve long-term survival [8–10]. Patients with advanced HCC which is considered as incurable have limited treatment options and chemotherapy provides minimal clinical benefit. Currently, Sorafenib, Lenvatinib, Regorafenib, or Opdivo, which are multitargeted kinase inhibitors or immune checkpoint inhibitors (Opdivo), are the only systemic agents demonstrated to extend overall survival (OS) compared with placebo in patients with advanced HCC by approximately three months [12–14].

Although TACE is relatively safe, it may cause liver damage complications, especially in presence of PVT [15]. In addition, like many cancers, HCC is also associated with hemostatic activation, with a reported incidence of PVT ranging from 20%-65% [16]. The presence of PVT in patients with HCC is associated with systemic VTE, worse hepatic function, intraarterial tumor invasion, portal hypertension, and poorer tolerance to undergoing treatment which are collectively lead to reduced survival [16, 17]. Not surprisingly, PVT is frequent in patients with liver cirrhosis which can be life-threatening [18]. Hemostatic alterations are well documented in liver disease: hemostasis is often impaired by thrombocytopenia and the reduced synthesis of coagulation factors that normally takes places in the liver [19]. These alterations can be worsened following surgery and chemotherapy even causing hemorrhagic complications [20, 21]. Thus, pharmacological prophylaxis of VTE is often needed in HCC patients. Despite the clinical relevance of the matter, there are no guidelines available on the administration of anti-thromboembolic prophylaxis in HCC patients. Currently, LMWH is strongly recommended for intervention in the prevention and management of thromboembolism complications [21–23]. Strikingly, several clinical and experimental studies have suggested that heparin derivatives affect cancer progression independent of their anticoagulant effects. Considering the fact that heparin derivatives are involved in a wide variety of biological activities, their application as an antithrombotic along with conventional therapy regime should be carefully planned to develop the best management strategies.

In this review, we firstly briefly review clinical applications of heparin derivatives in the management of cancer with a particular focus on HCC. Then we summarize the state of knowledge whereby heparin can cross-talk with molecules playing a role in hepatocarcinogenesis. Lastly, we highlight new experimental and clinical research conducted with the aim of moving towards personalized therapy in cancer patient at risk of thromboembolism.

## 2. Heparin Derivatives in the Management of Cancer

As mentioned above, patients with cancer are frequently treated with anti-coagulants, such as heparins, to treat or prevent thrombosis. Heparins are not absorbed orally, thus, they must be administrated parentally by intravenous infusion or subcutaneous injections [24]. UFH and other LMWHs, such as fondaparinux and danaparoid, do not possess intrinsic anticoagulant activity but potentiate antithrombin III that inhibits activated coagulation agents [25]. For many years unfractionated heparin (UFH) has been the standard treatment for initial anticoagulation [26]. However, recent randomized trials have demonstrated that LMWH is possibly superior to UFH in the initial treatment of VTE in people with cancer [26]. Moreover, LMWH provide other advantages versus UFH, including lower cost and simple dosing, and is associated with a lower risk for heparin-induced thrombocytopenia (HIT) [27]. Thus, LMWH is strongly recommended for intervention in the prevention and management of thromboembolism complications in cancer [21–23]. However, long-term use has been associated with bruising at injection sites, recurrent thromboembolism, thrombocytopenia, and bleeding which then causes interruption of essential cancer therapies [22, 26].

Importantly, randomized trials comparing LMWH to UFH for the treatment of thrombosis have also indicated that heparins may improve outcomes of patients with cancer, particularly in those with early stage disease cancer including HCC and in patients with small cell lung [28, 29]. The PROTECHT study (Prophylaxis of Thromboembolism during Chemotherapy; ClinicalTrials.gov Identifier: NCT00951574) has been designed to evaluate if prophylaxis with nadroparin (LMWH) conferred any additional benefit in terms survival, depending on whether chemotherapy disease control was achieved [30]. Notably, a statistically significant interaction between nadroparin treatment and response to chemotherapy was found, thus supporting the hypothesis difference in survival depends on the response to chemotherapy and nadroparin [30]. LMWHs lend themselves to such studies because of their pleiotropic effects and the relative ease of administration compared to UFH. A completed clinical trial, the results of which have not yet been released, investigated whether addition of LMWHs to TACE would improve HCC patient compared with TACE alone (ClinicalTrials.gov Identifier: NCT00827554). In addition, a current trial, which is not recruiting yet, aims to examine antithrombotic therapy with TACE in HCC to minimize mortality and to improve survival rate without provoking excessive bleeding (ClinicalTrials.gov Identifier: NCT02715492).

## 3. Heparin Derivatives

Heparin is a glycosaminoglycan that is synthesized by mast cells and basophils. Glycosaminoglycans are linear carbohydrate polymers that are composed of alternating uronate and hexosamine saccharides linked by glyosidic linkages [31]. Heparin undergoes extensive sulfation and rarely phosphorylation or carboxylation during synthesis and hence is a highly negatively charged biological molecule [32]. UHF is a naturally occurring mixture of glycosaminoglycan chains from porcine or bovine origin, each consisting of 200-300 saccharides units with molecular weights in the range of 12-14 kDa [33, 34]. LMWH consists of smaller fragments of UFH (nearly 18 saccharide units long, molecular weight approximately 5 kDa) produced by controlled enzymatic or chemical depolymerisation [33, 34]. Due to their structural differences, LMWH have relatively little antithrombotic activity compared to UFH [32].

## 4. Brief Review for Mechanisms of Heparin Affects Cancer Pathways

There are multiple experimental studies supporting the hypothesis that cancer progression can be influenced by heparins. Several* in vitro* and* in vivo* cancer models supported the idea that cancer cells exploit the coagulation system to facilitate cell growth, angiogenesis, immune evasion, and metastasis formation, by distinct mechanisms. In addition, numerous studies have demonstrated that heparins do not affect cancer only by their interaction with the coagulation cascade but also by various other ways, including by inhibition of cell-cell interaction by blocking cell-adhesion molecules (selectins), the inhibition of extracellular matrix proteinase heparanase, and the inhibition of angiogenesis.

Heparins are located primarily in the cell membrane and the extracellular matrix (ECM). They bind transiently with ECM-associated molecules, like the growth factors, cytokines, and enzymes, and alter their organization and functions [35]. One such example is heparin involvement in vascular epithelial growth factor- (VEGF-) fibronectin binding, where transient interaction of heparin with fibronectin promotes an open-conformation of fibronectin, which enhances binding to VEGF [36]. Consequently, VEGF binding to fibronectin is sufficient to mediate VEGF induced Erk1/2 activation, endothelial cell proliferation, and migration which are key steps in angiogenesis [37]. Moreover, blockage of the interaction between heparin and fibroblast growth factor (FGF), a well-known stimulator of angiogenesis, inhibits angiogenesis, tumor growth, and metastasis [38]. Duckworth et al. have shown that chemically modified heparin inhibits galectin-3-ligand binding and thereby prevents cancer-cell-endothelial adhesion and angiogenesis [39]. Also, heparin enhances ECM remodeling through the activation of metalloproteinase-2 and acts as a heparanase inhibitor that results in* in vitro* tubular morphogenesis of microvessels that is necessary for angiogenesis [33, 40]. Similarly, chemically modified nonanticoagulant species of heparin that specifically inhibit selectin-mediated heparanase enzymatic activity attenuate metastasis of melanoma cells [41]. It has been reported that heparin binds to platelets via P-selectin and prevents tumor invasion in lung cancer cells [42] Borsig et al. reported that heparin blocks P-selectin based platelet interactions through cell surface proteins such as mucins and thereby attenuates metastasis [42]. Furthermore, heparin binds L- and P-selectins to inhibit acute inflammation and thereby suppresses inflammatory processes in tumor microenvironment which is important for immune evasion. Moreover, heparin can also inhibit fibrin deposition around tumor cells for protecting cells from the immune system [43].

Another heparin interacting protein is factor 4 (CXCL4-PF4) which is released from activated platelets during platelet aggregation and promotes blood coagulation [44]. In addition, CXCL4-PF4 induces immune cells activation, differentiation, and migration but also inhibits endothelial cell migration, proliferation, and angiogenesis [44]. Relevant for this review are the effects of CXCL4-PF4 on the hemostatic system [44]. CXCL4-PF4 and heparin binding neutralise heparin on the endothelial surface of blood vessels, thereby inhibiting local antithrombin III activity and promoting coagulation [44]. In some patients exposed to heparin, CXCL4-PF4/heparin complex triggers an immunogenic response eventually leading to production of anti-CXCL4-PF4/heparin antibodies [44]. This may lead to a severe clinical condition characterized by platelet activation and aggregation, thrombocytopenia, and thrombocytopenia, which is commonly called heparin-induced thrombocytopenia (HIT). HIT typically develops 5-14 days after exposure to prophylactic or therapeutic doses of heparin [45]. In some cases, patients who previously had been exposed to heparin trigger HIT quicker [45].

In contrast to the anticarcinogenic effect above, heparin may also be involved in the activation of the metastatic cascade by forming a complex with midkine (MK). MK is highly expressed in HCC and in cancers of the stomach, colon, esophagus, pancreas lung, neuroblastoma, glioma, and urinary bladder [46–48]. MK is activated when it forms homodimers that are stabilised by heparin. Activated MK/heparin complex leads to metastasis and drug resistance [49]. Notably, Jia et al. demonstrated that LMWH significantly blocked coadhesion between connective tissue growth factor/CCN family 2 (CCN2) and low-density lipoprotein receptor-related protein 6 (LRP6) and enhanced chemotherapeutic effect of oxaliplatin on HCC [50]. CCN2 functions to orchestrate LRP6 which is coreceptor in Wnt signalling. Wnt signalling is key signaling related to stem-ness, and chemoresistance [50]. Combination treatment with oxaliplatin and LMWH showed improved response rates to chemotherapy [50] Likewise, Pfankuchen et al. reported that a therapeutic dosage of LMWH (tinzaparin) reversed cisplatin resistance in a clone of ovarian cancer cell line to the level of sensitive cells [51, 52]. According to the follow up study, cisplatin resistant cells showed 3-fold higher Wnt signaling activity compared to wild type cells and Wnt pathway blockade increased cisplatin sensitivity. LMWH treatment reduced Wnt pathway activity and TCF-4 expression and enhanced cisplatin sensitivity in cisplatin resistant clones [51].

LMWH is also used as a nanocarrier to deliver drugs in cancer therapies. Modification of heparin molecules to generate nanocarriers become useful for applications like imaging, disease, and cancer treatments. Yan et al. prepared gambogic acid grafted low molecular weight heparin micelles to combine anti-tumor effect of gambogic acid with anti-angiogenic and anti-metastatic effect of heparin [53]. This* in vivo* study suggested that drugs grafted to LMWHs can be delivered to the liver and enhance their therapeutic effects by combining antitumor effects of heparin [53]. Furthermore, Du et al. modified LMWH to carry doxorubicin to overcome doxorubicin resistance in HCC [54].

## 5. Factors Influencing the Pleiotropic Role of Heparins

**Figure 1:**
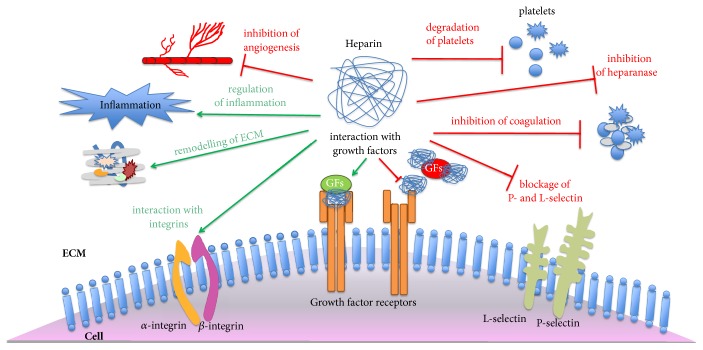
**Biological effects of heparin**. Heparin has inhibitory and activating roles in molecular and cellular mechanisms. It has a role in degradation of platelets, inhibition of coagulation, and angiogenesis. It also acts as a heparanase inhibitor and blocks P- and L-selectin to interact with platelets and prevents metastasis. Moreover, it interacts with ECM proteins and enhances remodeling of the ECM. It is involved in inflammatory processes and regulates inflammation. Heparin interacts with integrins and growth factors. However, in some growth factor signaling pathways it may have inhibitory as well as activating effects. For instance, it interacts with factors such as FGF, TGFB1, and MK and regulates the signaling positively. In contrast, it also interacts with FGF and HGF and regulates the signaling negatively (ECM: extracellular matrix; FGF: fibroblast growth factor; and HGF: hepatocyte growth factor.

Due to their heterogeneity and natural location, heparins are able to interact with a wide variety molecules and mediate diverse biological processes (Figure 1). Therefore, it is not surprising that the role of heparins in tumor genesis is context dependent.

To clarify the various potential mechanisms of heparin anticancer activity, Niers et al. evaluated the data from preclinical studies (published between 1960 and 2005) in which heparins have been tested as anticancer therapy [32]. They suggested that heparin may affect the formation of metastasis rather than the growth of primary tumors. They also documented that chemically modified heparins with no or limited anticoagulant activity also showed antimetastatic properties [32]. They concluded with possible mechanisms to explain the effects on the process of metastasis include inhibition of blood coagulation, inhibition of cancer cell-platelet, and cancer cell-endothelial interactions by inhibition of cell invasion and angiogenesis [32]. They also documented interesting results highlighting the importance of types, duration, timing, and dose of heparin used, animal tumor model tested, and route of heparin administration in the course of disease [32]. Similarly, LMWH treatment was shown to inhibit FGF-induced mitogenesis of tumor derived endothelial cells in a time and concentration dependent manner [55]. Likewise, in Jha et al.'s study, to understand the effects of molecular weight and concentration of heparin on transforming growth factor (TGF)-*β*1 signaling, they used heparin-containing hyaluronic acid based hydrogels to analyze growth factor affinity and retention [56]. At equal concentrations, high molecular weight heparin has the highest amount of TGF-*β*1 retention from hydrogel compared to low molecular weight or unfractionated heparin. This response is critical for stem cell differentiation and lineage specification [56]. LaRochelle et al. determined that low concentrations of heparin enhance the binding of keratinocyte growth factor (KGF) to its receptor in CHO cells lacking HS proteoglycans but this effect is not observed in wild type CHO cells. In contrast higher heparin concentrations inhibit KGF signaling [57]. Furthermore, while heparins with short chain lengths are not able to activate anaplastic lymphoma kinase (ALK), heparins with longer chain lengths can induce dimerization and activation ALK in neuroblastoma cells [58]. Interestingly, in our previous studies, we showed that heparin can activate the c-Met signaling pathway by activating dimerization of c-Met receptor, which can then induce HCC cell invasion [59] However, when Hepatocyte Growth Factor (HGF), ligand of c-Met, is in the environment, heparin suppressed HGF/c-Met signaling mediated adhesion, motility, and invasion [59]. When we performed a microarray analysis to identify the molecular mechanisms behind heparin mediated biological responses, we observed that heparin modulates transcription of several genes involved in glucose metabolism, tumor angiogenesis, and EMT [60]. In our further analysis, we demonstrated that heparin controlled thioredoxin-interacting protein (TXNIP) gene expression through two mechanisms: (1) it can either directly bind to a unique carbohydrate response element located on the promoter of this gene or (2) it can trigger epigenetic modifications [60]. In either case, increased expression of TXNIP, which is a regulator glucose metabolism, accelerates migration and invasion abilities of HCC cells [60]. These data imply that, in addition to its regulatory role on receptor dimerization and ligand binding to its receptor, heparin also has a transcription regulatory role in HCC.

**Figure 2:**
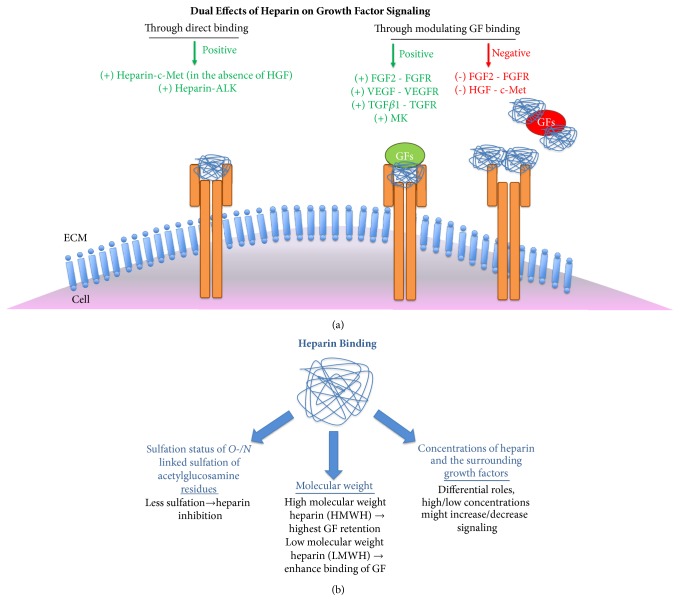
**Effects and effectors of heparin binding**. Heparin binding has context-dependent roles in Growth Factor (GF) signaling, it might directly bind to Growth Factor Receptors (GFRs) or it might modulate signaling positively or negatively through binding to both GFs and GFRs (a). Heparin binding to GFs and GFRs are affected by several factors including sulfation status, the molecular weight of heparin and the concentrations of both heparin and the GFs (b).

Overall, these studies emphasised that both intrinsic (such as tumor type) and extrinsic (such as heparin type) determinants play roles in the actions of heparin on tumors. For instance, while heparin administration increases tumor growth and metastasis in colon cancer, it reduces metastases in fibrosarcomas, lung, prostate, and mammary carcinomas [61–63]. These pleotropic effects of heparin might be related to expression or activation levels of growth factors and/or their receptors, as well as by heparin type and concentration. As summarized in Figure 2, heparin can directly bind to growth factor receptors or growth factors to stimulate signaling pathways, whereas it could block growth factor-receptor interaction as a context dependent manner.

## 6. Conclusions

The management of HCC along with other advanced-stage cancers remains a challenge. Venous thrombosis is a common complication in patients with cancer and indicates a poor prognosis. LMWH is widely used in the clinic as an anticoagulant as part of a treatment regimen in cancer patients to treat or control thrombosis. Many studies highlight the benefit of heparin derivatives in increasing patient survival, mostly through their antithrombotic effect. There is also a growing amount of evidence for the anticancer effects of heparin, which are mostly via its inhibition of metastasis rather than on primary tumor growth. However, there is also evidence revealing that heparin can act as a metastasis promoting agent. Clearly, due to its pleiotropic actions, heparin affects tumorigenesis in many ways and may promote or hamper cell transformation, depending on the cancer type and stage along with its structural properties and concentration. This phenomenon stresses the fact that heparin use in the clinic should be assessed carefully. In addition, the use of a same therapy approach for all patients might result in variable and unpredictable responses, because of heterogeneity among tumors and genotypic differences between patients. Hence, personalized medicine (PM) offers an attractive approach for cancer management and care. PM implements “-omic" sciences (genomics, epigenomics, transcriptomics, proteomics, etc.) to integrate various data sets with the aim of dissecting molecular signatures and functional pathways that help to classify tumor subtypes and determine their natural course, prognosis, and responsiveness to therapies [64]. Thus, stratification of the subset of patients who might respond to particular combinations of therapies is crucial in the management of cancer. For instance, mutations of the KDM6A, CUL9, FDG6, AKAp3, and RFN139 genes are associated with the development of PVT in advanced HBV-related HCC [65]. Since effective management of PVT may improve treatment results for HCC, these genes can be used for the identification or prediction of high-risk patients who will benefit most from antithrombotic therapy. Moreover, anti-CXCL4-PF4/heparin antibodies can be used as a predictive factor to identify patients who should avoid heparin treatment. Further studies are needed for better understanding of heparin and tumor biology and the determination of biomarkers for the planning of best evidence-based approaches that meet the needs of patients for disease treatment, reduction of symptoms, and improvement in quality of life.
